# A Computer Simulation of SARS-CoV-2 Mutation Spectra for Empirical Data Characterization and Analysis

**DOI:** 10.3390/biom13010063

**Published:** 2022-12-28

**Authors:** Ming Xiao, Fubo Ma, Jun Yu, Jianghang Xie, Qiaozhen Zhang, Peng Liu, Fei Yu, Yuming Jiang, Le Zhang

**Affiliations:** 1 College of Computer Science, Sichuan University, Chengdu 610065, China; 2 Med-X Center for Informatics, Sichuan University, Chengdu 610041, China; 3 West China Biomedical Big Data Center, West China Hospital, Sichuan University, Chengdu 610041, China; 4 CAS Key Laboratory of Genome Sciences and Information, Beijing Institute of Genomics, Chinese Academy of Sciences, Beijing 100049, China; 5 College of Life Sciences, University of Chinese Academy of Sciences, Beijing 100049, China; 6 National Wildlife Health Center, Hebei Agricultural University, Baoding 071001, China; 7 Hebei Key Laboratory of Analysis and Control of Zoonotic Pathogenic Microorganism, Hebei Agricultural University, Baoding 071001, China; 8 College of Life Sciences, Hebei Agricultural University, Baoding 071001, China

**Keywords:** SARS-CoV-2, mutation spectra, mutation simulation, computational biology, bioinformatics

## Abstract

It is very important to compute the mutation spectra, and simulate the intra-host mutation processes by sequencing data, which is not only for the understanding of SARS-CoV-2 genetic mechanism, but also for epidemic prediction, vaccine, and drug design. However, the current intra-host mutation analysis algorithms are not only inaccurate, but also the simulation methods are unable to quickly and precisely predict new SARS-CoV-2 variants generated from the accumulation of mutations. Therefore, this study proposes a novel accurate strand-specific SARS-CoV-2 intra-host mutation spectra computation method, develops an efficient and fast SARS-CoV-2 intra-host mutation simulation method based on mutation spectra, and establishes an online analysis and visualization platform. Our main results include: (1) There is a significant variability in the SARS-CoV-2 intra-host mutation spectra across different lineages, with the major mutations from G- > A, G- > C, G- > U on the positive-sense strand and C- > U, C- > G, C- > A on the negative-sense strand; (2) our mutation simulation reveals the simulation sequence starts to deviate from the base content percentage of Alpha-CoV/Delta-CoV after approximately 620 mutation steps; (3) 2019-NCSS provides an easy-to-use and visualized online platform for SARS-Cov-2 online analysis and mutation simulation.

## 1. Introduction

SARS-CoV-2 [1], discovered in 2019, is an RNA coronavirus with a positive-sense single-stranded genome, which repeatedly replicates and mutates within host cells by continuously changing the underlying molecular structure [2]. Currently, it is a global pandemic disease with a great social and economic impact on people worldwide. Especially, the gradual accumulation of mutations could generate new viral variants, leading to the failure of corresponding vaccines and diagnostic therapies [3,4]. As an effective virus research method, computer simulation could help us predict SARS-CoV-2 outbreak, make virus traceability, and design vaccine and drug by computing the viral mutation spectra [5] from sequencing data to describe the relative frequencies of all base mutation types and simulate the mutation process of SARS-CoV-2 based on the mutation spectra [6]. 

In terms of SARS-CoV-2 intra-host mutation spectra computation, although recent studies already analyzed the intra-host mutation spectra of SARS-CoV-2 [7,8], they neither employed dynamics thresholds to filter low-quality data during raw sequencing data processing nor considered data specificity. Furthermore, since most of these computational methods are based on next-generation sequencing data, which is inherently deficient in short read-length and the need of amplification, thus it is difficult to accurately distinguish positive- and negative-sense sub-genomes or to further investigate the strand specificity of SARS-CoV-2 intra-host mutants [5,9]. Therefore, our first scientific question is how to develop such a data specificity-based base filtering algorithm for SARS-CoV-2 sequencing data that can significantly improve the accuracy of strand-specific mutation spectra computation for SARS-CoV-2.

In terms of SARS-CoV-2 mutation simulation, many researchers have carried out mutation simulation for SARS-CoV-2 from the perspective of viral genomics. For example, Hurst et al. [10] investigated the genetic specificity of CG content percentage and CpG dinucleotides among the whole genome sequences of existing SARS-CoV-2 to infer the pattern of mutations. Additionally, Rosset et al. [11] developed a statistical model based on generalized linear model (GLM) to describe the replacement process of SARS-CoV-2 and predict its future mutations. However, since these previous works [10,11] seldom integrate the mutational characteristics of coronavirus into actual mutation spectra, it is very inefficient and time consuming to investigate the important characteristics of sequence during the mutation process [11]. Therefore, our second scientific question is how to develop an efficient SARS-CoV-2 intra-host mutation simulation and data analysis algorithm with respect to the intra-host mutation spectra and coronavirus mutation characteristics.

Meanwhile, although many online databases and services related to SARS-CoV-2 have been established [12,13], most of them only focused on the statistical analysis and the viral genomic data download rather than provide functions such as SARS-CoV-2 mutation spectra analysis or mutation simulation. Therefore, our third scientific question is how to build up a visualized web service platform for online mutation spectra analysis and mutation simulation. 

Here, we propose three major innovations to answer the above scientific questions. Firstly, we propose a computational analysis algorithm for SARS-CoV-2 genomic mutation spectra based on nanopore sequencing [14], which not only utilizes a nucleobase filtering algorithm with dynamic threshold, but also takes the advantages of nanopore sequencing in long read-length and amplification-free [14,15] to accurately distinguish positive- and negative-sense sub-genomes. 

Secondly, we build up a Markov chain-based intra-host mutation simulation and data analysis process with respect to the SARS-CoV-2 mutation spectra, which can not only improve the simulation efficiency by exploring the convergence interval of the mutation simulation model, but also analyze the different sequence properties such as base content percentage, the distribution of stop codons and sequence periodicity during mutation process.

Finally, we establish an online service platform for SARS-CoV-2 mutation spectra analysis and mutation simulation, which not only provides researchers with data downloading, online computational analysis and visualization services, but also allows validation and feedback optimization of the mutation simulation process with continuously accumulated data.

Based on the above innovations, we present a strand-specific intra-host mutation spectra computation algorithm of the SARS-CoV-2 genome and compute the intra-host mutation spectra of positive- and negative- sense strands within different lineages of SARS-CoV-2. Afterwards, based on the intra-host mutation spectra, we develop a novel Markov chain-based intra-host mutation simulation model and analyze the changes of sequence properties of the SARS-CoV-2 genome during the mutation simulation. Finally, we established a web service platform based on the mutation spectra computation method and simulation model.

Our main results include: (1) there is a significant variability in the SARS-CoV-2 intra-host mutation spectra across different lineages, with the major mutations from G- > A, G- > C, G- > U on the positive-sense strand and C- > U, C- > G, C- > A on the negative-sense strand; (2) our mutation simulation reveals that the simulation sequence starts to deviate from the base content percentage of Alpha-CoV/Delta-CoV after approximately 620 steps of mutation, which is not only consistent with previous studies in which a cell generates approximately 600 to 700 viral particles on average [16], but also demonstrate the validity of the mutation simulation method; and (3) our website provides an easy-to-use and visualized online platform for SARS-CoV-2 mutation spectra analysis and mutation simulation.

## 2. Materials and Methods

This study employs the SARS-CoV-2 genome MT039890 [17] from the NCBI database as the reference genome sequence. Also, the SARS-CoV-2 nanopore sequencing data are downloaded from the NCBI GEO [18] database, which includes 22 sequencing projects in total. Each sequencing record consists of sequence identifier, sequence, and base quality scores, which is detailed by Table S1. 

**Figure 1:**
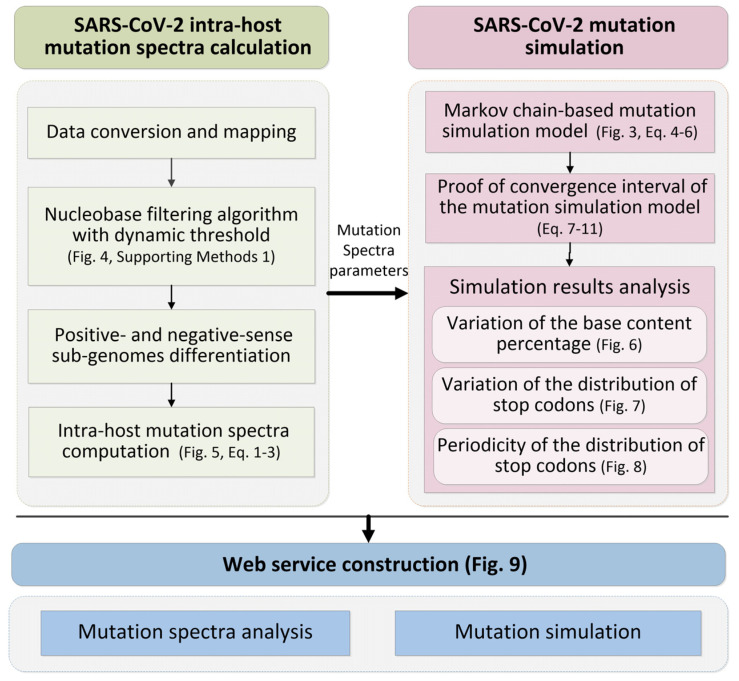
The workflow of the study.

Figure 1 describes the workflow of the study with three main steps: SARS-CoV-2 intra-host mutation spectra computation (left side of Figure 1), SARS-CoV-2 mutation simulation (right side of Figure 1) and web service construction (bottom of Figure 1).

In the SARS-CoV-2 intra-host mutation spectra computation step (Figure S1), we first map the SARS-CoV-2 nanopore sequencing data onto the reference genome. Second, we propose a novel dynamic threshold-based base filtering algorithm to efficiently filter the data with poor sequencing quality. Afterwards, to further investigate the strand specificity of SARS-CoV-2 intra-host mutants, we distinguish the positive- and negative-sense sub-genomes sequencing data. Finally, we compute the SARS-CoV-2 intra-host mutation spectra of positive- and negative-sense sub-genomes, respectively. 

In the SARS-CoV-2mutation simulation step, we first propose a Markov chain-based intra-host mutation simulation model using the SARS-CoV-2 mutation spectra as key parameters. Second, to improve the simulation efficiency of the model, we prove the convergence of the mutation simulation model and find the minimum number of repetitions of the simulation. Finally, we analyze the results of mutation simulation, including the base content percentage, the distribution of stop codons and sequence periodicity during mutation process. 

In the Web service construction step, based on the methods and results of mutation spectrum computation and mutation simulation, we establish an online service platform for SARS-CoV-2 mutation spectra analysis and mutation simulation. The specific methods are described as follows.

### 2.1. SARS-CoV-2 Intra-Host Mutation Spectra Computation

#### 2.1.1. Data Conversion and Mapping 

First, we decompressed the SRA format data [19], then converted it to Fastq format [20], and mapped it onto the SARS-CoV-2 reference genome sequence by Minimap2 [21]. 

#### 2.1.2. Nucleobase Filtering Algorithm with Dynamic Threshold 

**Figure 2:**
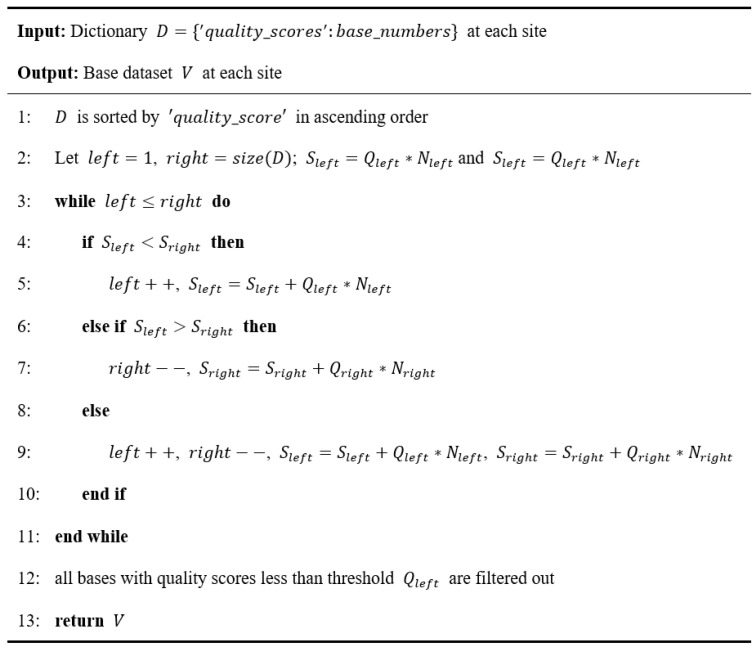
The pseudo code of nucleobase filtering algorithm with dynamic threshold. Here, D represents the dictionary which stores ‘quality_scores’:base_numbers pairs at each site; V represents the base dataset after filtering by the algorithm at each site; Q represents the base quality score corresponding to left or right; N represents the base number corresponding to $Q_{left}$ or $Q_{right}$. We implement the algorithm for the mapped sequencing reads at each site of the reference genome to obtain the high-quality sequence data for mutation spectra analysis.

To replace the fixed threshold during data filtering in the previous algorithms [22,23,24], we develop a nucleobase filtering algorithm with dynamic threshold. The algorithm filters the low-quality base qualities during data mapping to determine the dynamic threshold interval by calculating the distribution specificity of sequencing data through the moving of pointers, which are detailed by Figure 2.

#### 2.1.3. Positive- and Negative-Sense Sub-Genomes Differentiation 

Based on the replication principle of SARS-CoV-2 [25], we use Samtools [26] to differentiate positive- and negative-sense sub-genomes. By setting the parameter “-F 16”, Samtools can efficiently specify the filtered sequencing data which reversely complemented with the positive-sense reference genome sequence as the negative-sense strand.

#### 2.1.4. Intra-Host Mutation Spectra Computation 

First, we use Equation (1) to compute the probability of 12 base mutation types at each site of the genome.

(1) $$\begin{array}{c} p_{site}\left(r\to m\right)=\frac{n\left(m\right)}{n\left(r\right)+n\left(m\right)} \end{array}$$

Here, $n\left(r\right)$ represents the number of bases r with the greatest number of reads at each site and $n\left(m\right)$ represents the respective number of other three bases m which excludes r at each site. r→m represents the mutation from base r to base m. $r,m\in \left\{A,U,C,G\right\}$, and r≠m.

Second, we use Equation (2) to compute the initial probability of the 12 base mutation types on the genome.

(2) $$\begin{array}{c} \;p_{genome}\left(r\to m\right)=\frac{\sum_{site\;}p_{site}\left(r\to m\right)}{n\left(r\to m\right)} \end{array}$$

where $p_{site}\left(r\to m\right)$ represents the probability of base mutation type r→m at each site, $n\left(r\to m\right)$ represents the number of sites where base mutation type r→m occurs.

Finally, the results from Equation (2) are normalized by Equation (3) to obtain the intra-host mutation spectra of the SARS-CoV-2.

(3) $$\begin{array}{c} \;\bar{p_{genome}\left(r\to m\right)}=\frac{p_{genome}\left(r\to m\right)}{\sum_{r\to m}p_{genome}\left(r\to m\right)} \end{array}$$

where $p_{genome}\left(r\to m\right)$ represents the initial probability for each base mutation type r→m, and $\underset{r\to m}{\sum }p_{genome}\left(r\to m\right)$ represents the sum of all 12 initial probabilities for each base mutation type r→m.

### 2.2. SARS-CoV-2 Mutation simulation

#### 2.2.1. Markov Chain-Based Mutation Simulation Model

##### 2.2.1.1. Sequences Data Preprocessing

**Figure 3:**
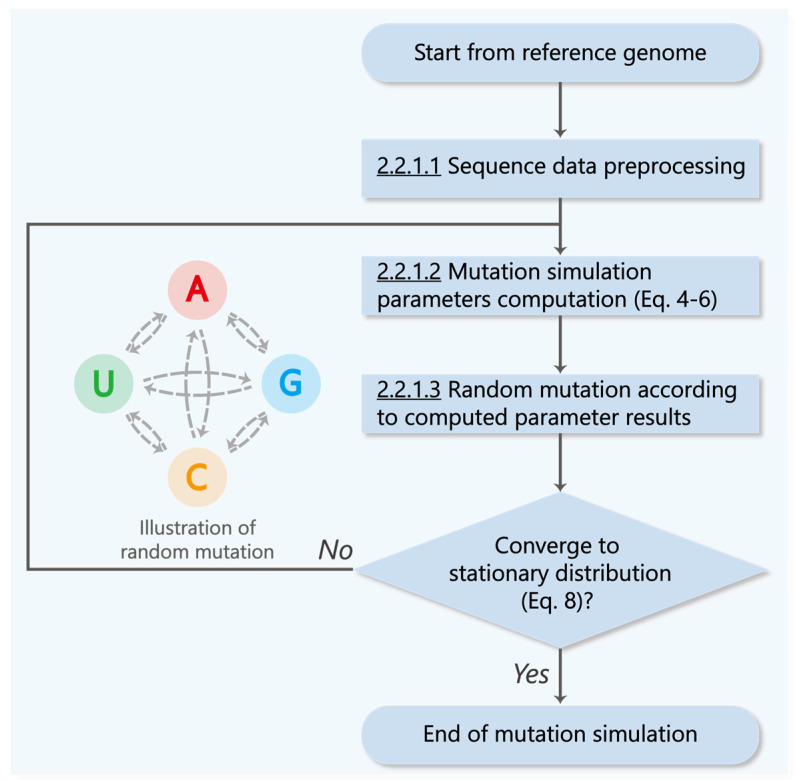
The mutation simulation flowchart of SARS-CoV-2.

Figure 3 describes our developed Markov chain-based mutation simulation model. Since we focus on the mutations of the coding sequences, the first procedure is to remove the non-coding sequences UTR and Intergenic from the reference genome [17], and the remaining sequences are used as the initial sequence for the mutation simulation.

##### 2.2.1.2. Mutation Simulation Parameters Computation

The second procedure is to use the mutation spectra that computed from real data as parameters to simulate one step of the replication mutation process of coronavirus. The mutation sites are randomly selected according to the proportion of mutations of different base types (Equation (4)). The site number n for each mutation is computed by Equation (5). The random probability of mutation direction for each site is determined by the mutation probability matrix (Equation (6)) with respect to the mutation spectra.

(4) $$\begin{array}{c} \;r_{i}=\frac{n_{i}}{n_{all}},i\in \left\{A,U,C,G\right\} \end{array}$$

Here, $r_{i}$ represents the proportion of mutations of base type i, $n_{i}$ represents the site number of mutations in base type i, and $n_{all}$ represents the number of all bases in which mutations occur.

(5) $$\begin{array}{c} n=\lfloor \mathrm{mutation}\text{\_}\mathrm{rate}\times geo\_len\rfloor \end{array}$$

Here, mutation_rate represents the viral mutation rate, namely the substitution frequency of each nucleotide per replication round, and geo_len represents the total genome length of SARS-CoV-2.

(6) $$\begin{array}{cc} & \;\;\;\;A\;\;\;U\;\;\;\;\;C\;\;\;\;\;G\;\;\;\\ \begin{array}{c} A\\ U\\ C\\ G \end{array} & \left\{\begin{array}{cccc} r_{A}m_{A,A} & r_{A}m_{A,U} & r_{A}m_{A,C} & r_{A}m_{A,G}\\ r_{U}m_{U,A} & r_{U}m_{U,U} & r_{U}m_{U,C} & r_{U}m_{U,G}\\ r_{C}m_{C,A} & r_{C}m_{C,U} & r_{C}m_{C,C} & r_{C}m_{C,G}\\ r_{G}m_{G,A} & r_{G}m_{G,U} & r_{G}m_{G,C} & r_{G}m_{G,G} \end{array}\right\} \end{array}$$

Here, $m_{i,j}$ represents the mutation probability from base i to base j, it could be obtained from the mutation spectra. When i equals to j, it represents the probability that no base mutation occurs.

##### 2.2.1.3. Random Mutation according to Computed Parameter Results

According to the computed parameter results from Equations (4)–(6), the mutation simulation is repeated until the genomic components converge to a stationary distribution (details in Section 3.2.2), by which we simulate the cumulative mutation process of coronavirus. Since the mutation probability of sequence sites are fixed and it can be looked as a stochastic process without posteriority, we can consider it as a Markov process [27].

#### 2.2.2. Proof of Convergence Interval of the Mutation Simulation Model

Here, we define each site of the Markov chain as $\left\{X_{t},\;t\geq 0\right\}$, where $X_{t}$ represents the simulation state of the system for each site at each time point t=0,1,2,…. The set of possible base type values, $\left\{A,\;U,\;C,\;G\right\}$, of $X_{t}$ is the possible state of the system. We define the mutation probability matrix of $X_{t}$ as $P_{i,j}$, which represents if current system state is base i, then it will have $P_{i,j}$ probability to be the next system state as base j. 

Equation (6) describes the mutation probability matrix ($P_{i,j}=${$p_{i,j}$}), $p_{i,j}=r_{i}m_{i,j}\;i,j\in \left\{A,\;U,\;C,\;G\right\}$. Since $X_{t}$ must be in a specific base after it leaves base i, $p_{i,j}$ satisfy Equation (7) [28].

(7) $$\begin{array}{c} \;\sum_{j}p_{i,j}=1,i,j\in \left\{A,\;U,\;C,\;G\right\} \end{array}$$

Since Equation (6) shows that for all base i and j, $p_{i,j}>0$, thus $X_{t}$ is irreducible [29]; Also, for all base i that $p_{i,i}>0$, thus $X_{t}$ is aperiodic [29]. Therefore, $X_{t}$ has a unique solution of the stationary distribution. We introduce the mutation spectra parameter in order to estimate the mutation steps when the initial distribution of $X_{t}$ converge to its stationary distribution $\pi \left(\cdot \right)$ by satisfying Equation (8).

(8) $$\begin{array}{c} \;\pi \left(\cdot \right)P_{i,j}=\pi \left(\cdot \right) \end{array}$$

where $\pi \left(\cdot \right)$ is a row vector and $\sum \pi \left(\cdot \right)=1$. We explore how many mutation steps will converge by computing the information entropy $H\left(x\right)$ with Equations (8) and (9).

(9) $$\begin{array}{c} \;H\left(x\right)=-\sum_{i}g\left(x_{i}\right)\log\left(g\left(x_{i}\right)\right) \end{array}$$

Here, x is the gene sequence. And $x_{i}$ and $g\left(x_{i}\right)$ are the base and the proportion of $x_{i}$ in x, respectively. Next, Section 3.2.1 will detail the specific computational procedure and results.

## 3. Results

### 3.1. SARS-CoV-2 Intra-Host Mutation Spectra Computation

To answer our first scientific question, we develop a nucleobase filtering algorithm with dynamic threshold, and then compute the intra-host mutation spectra of SARS-CoV-2.

#### 3.1.1. Nucleobase Filtering Algorithm with Dynamic Threshold

**Figure 4:**
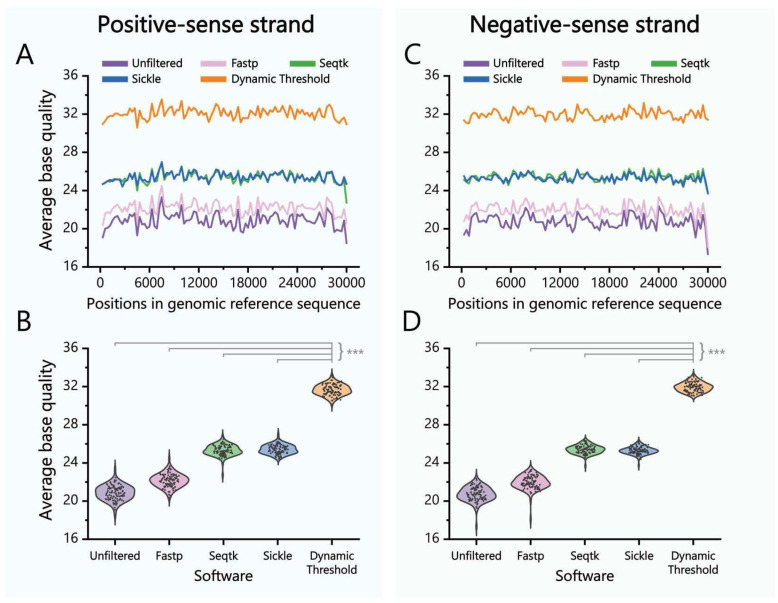
Comparing the low-quality data filtering results of five approaches by average base quality within each window length (300 bases). (**A**) The specific average base quality of SARS-CoV-2 positive-sense reference strand at each site. (**B**) The overall average base quality distribution of SARS-CoV-2 positive-sense reference strand at each site. (**C**) The specific average base quality of SARS-CoV-2 negative-sense reference strand at each site. (**D**) The overall average base quality distribution of SARS-CoV-2 negative-sense reference strand at each site. *** *p* ≤ 0.001.

We use an approach based on the comparison of average base quality to validate the advantage of nucleobase filtering algorithm with dynamic threshold over fixed threshold nucleobase filtering tools [30]. Here, we randomly choose a SARS-CoV-2 sequencing project, PRJNA610248 (Table S1), as the test case. After data preprocessing illustrated in Section 2.1, the positive- and negative-sense strand of this project that mapped on the reference genome include approximately 6,000,000 SARS-CoV-2 sequencing reads in total. Then, we carried out sequencing data filtering algorithms respectively with the following five approaches: unfiltered, fastp [22], seqtk [23], sickle [24] and the nucleobase filtering algorithm with dynamic threshold, which are detailed in Figure 2. Figure 4 shows the comparison results.

The test of significance [4,31,32,33,34,35,36,37,38] between the nucleobase filtering algorithm with dynamic threshold and the other four filtering methods are implemented, respectively, by Method S1. Figure 4A,B indicates that the average base quality of SARS-CoV-2 positive-sense reference strand processed by unfiltered, fastp, seqtk and sickle are mostly distributed between 18–26, while the average base quality processed by the nucleobase filtering algorithm with dynamic threshold are mostly distributed between 30–34. This result is consistent in negative-sense reference strand, as illustrated in Figure 4C,D. Therefore, the corresponding average base quality filtered by nucleobase filtering algorithm with dynamic threshold are statistically better than the other four methods (Tables S3 and S4).

#### 3.1.2. Intra-Host Mutation Spectra Computation

Figure 4 shows the SARS-CoV-2 intra-host mutation spectra of 22 projects computed from the filtered high-quality sequences by Equations (1)–(3). 

**Figure 5:**
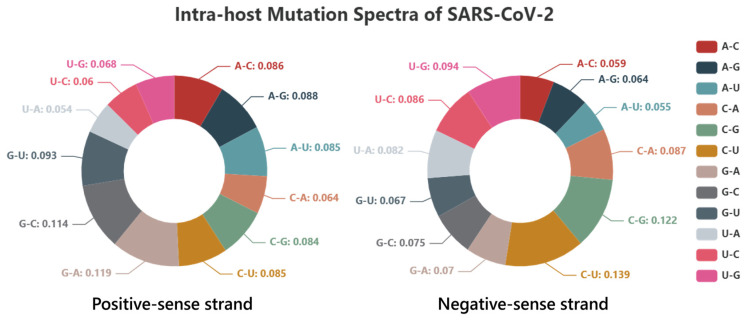
Intra-host mutation spectra of SARS-CoV-2. The size of each color in the pie chart indicates the proportion of the corresponding base mutation type within the intra-host mutation spectra.

After computing the histogram of SARS-CoV-2 strand-specific mutations probability (Figure S2), Figure 5 demonstrates that the intra-host mutation spectra of SARS-CoV-2 has a greater occurrence probability of mutation types G->A, G->C and G->U in the positive-sense strand, as well as C->U, C->G and C->A in the negative-sense strand. Separated intra-host mutation spectra of each of 22 projects can be obtained by referring to the website (http://www.combio-lezhang.online/2019NCSS/home.html accessed on 22 September 2022).

### 3.2. SARS-CoV-2 Mutation Simulation

To answer our second scientific question, we firstly prove the convergence of mutation simulation model to determine the simulation steps (Section 3.2.1). Then, we dynamically analyze the sequence properties such as base content percentage, the distribution of stop codons and sequence periodicity for the genomic sequences during the mutation simulation (Section 3.2.2, Section 3.2.3 and Section 3.2.4) by integrating the sequence properties of four SARS-CoV-2 lineages (including Alpha-CoV, Beta-CoV, Gamma-CoV and Delta-CoV) [32].

#### 3.2.1. Proof of Convergence Interval of the Mutation Simulation Model

To reduce the time consumption for mutation simulation, we mathematically prove the convergence interval of the mutation simulation model to determine the simulation steps.

We input our previous research (Table S5) [32] and the results of Equation (4) into Equation (6) to have the mutation probability matrix $P_{ij}$ (Method S2).

(10) $$\begin{array}{cc} & \;\;\;A\;\;\;\;U\;\;\;\;C\;\;\;\;G\;\;\;\\ \begin{array}{c} A\\ U\\ C\\ G \end{array} & \left(\begin{array}{cccc} 0.999897 & 0.000014 & 0.000011 & 0.000078\\ 0.000009 & 0.999897 & 0.000084 & 0.00001\\ 0.000004 & 0.000098 & 0.999897 & 0.000001\\ 0.000029 & 0.000069 & 0.000005 & 0.999897 \end{array}\right) \end{array}$$

The Markov chain represents the mutation process of the whole chain. When a site on the genome is *A*, *U*, *C* or *G*, the initial distributions of its each initial state is $\pi \left(A\right)=\left(1,0,0,0\right)$, $\pi \left(U\right)=\left(0,1,0,0\right)$, $\pi \left(C\right)=\left(0,0,1,0\right)$ or $\pi \left(G\right)=\left(0,0,0,1\right)$. Let the stationary distribution $\pi \left(\cdot \right)=\left(p_{A},p_{U},p_{C},p_{G}\right)$, and then we have Equation (11) by inputting the Equation (10) into Equation (8).

(11) $$\begin{array}{c} \;\left\{\begin{array}{c} 0.999897p_{A}+0.000009p_{U}+0.000004p_{C}+0.000029p_{G}=p_{A}\\ 0.000014p_{A}+0.999897p_{U}+0.000098p_{C}+0.000069p_{G}=p_{U}\\ 0.000011p_{A}+0.000084p_{U}+0.999897p_{C}+0.000005p_{G}=p_{C}\\ 0.000078p_{A}+0.00001p_{U}+0.000001p_{C}+0.999897p_{G}=p_{G}\\ p_{A}+p_{U}+p_{C}+p_{G}=1 \end{array}\right. \end{array}$$

Based on the detailed balance condition [39] of Markov chain and using the iterative solution method [40], we compute that the value of Equation (11) will converge to a stationary distribution as $\pi \left(\cdot \right)=\left(p_{A},p_{U},p_{C},p_{G}\right)=\left(0.08,0.44,0.11,0.37\right)$ after about 80,000 times of mutation simulation, which means it reaches a smooth distribution. Therefore, we set 80,000 as the upper limit for the number of simulations of the model.

Subsequently, we also computed the variation of information entropy [41] during simulation according to Equation (9) (Method, Figure S3). We find that the sequence mutation information entropy tends to be constant before 80,000 steps, which verifies the validity of the upper limit of the mutation obtained by Equation (11). Therefore, we increase the efficiency of the SARS-CoV-2 intra-host mutation simulation by determining the upper limit of the mutation.

#### 3.2.2. Variation of the Base Content Percentage

**Figure 6:**
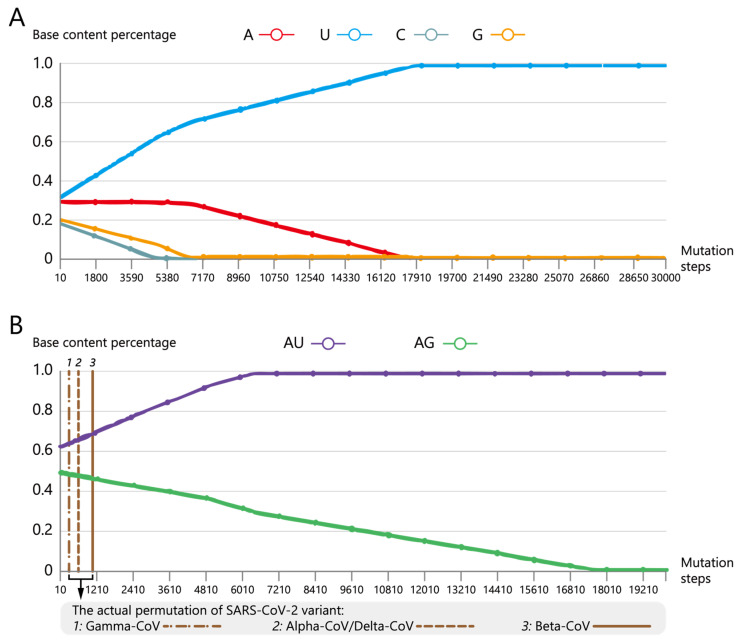
Variation of base content percentage in mutation simulation. (**A**) The percentage of four base content during mutation simulation. The horizontal axis represents the cumulative mutation number and the vertical axis represents the base percentage. Red, blue, dark green and orange lines represent the content percentage of A, U, C and G, respectively. (**B**) The percentage of “AG” and “AU” content during the mutation simulation. Brown dotted lines represent how many mutation steps that the percentage of “AG” and “AU” content would deviate from the base content percentage of Gamma-CoV, Alpha-CoV/Delta-CoV and Beta-CoV, respectively. Purple and light green lines represent the percentage of “AG” and “AU” content, respectively.

In Figure 6, we investigate the variation of each base content percentage during mutation simulation using the base content percentage of the reference genome as the initial state. Especially, we investigate the relationship between the critical interval of base content percentage and the actual permutation of the virus [27,32] by introducing the value “AG” (the sum of base content percentage A and G) and “AU” (the sum of base content percentage A and U) of four SARS-CoV-2 lineages [27] during simulation. According to the previous research [27], the base content range of four lineages are AU: 0.56–0.66, AG: 0.46–0.51 for Alpha-CoV, AU: 0.54–0.69, AG: 0.46–0.51 for Beta-CoV, AU: 0.53–0.66, AG: 0.46–0.51 for Delta-CoV, and AU: 0.60–0.66, AG: 0.47–0.52 for Gamma-CoV, respectively.

Figure 6A shows that the basic trend of mutation simulation is the increase of U and the decrease of C and G. C and G disappears almost completely after about 5000 and 6000 steps of mutation, respectively. After about 17,000 steps of mutation, almost the entire sequence mutates to U, and there is no obvious variation in base content percentage afterwards. In addition, Figure 6B shows that the simulation sequence starts to deviate from the base content percentage of Gamma-CoV (AU: 0.60–0.66, AG: 0.47–0.52) after about 300 steps of mutation; Subsequently, the simulation sequence starts to deviate from the base content percentage of Alpha-CoV and Delta-CoV (Alpha-CoV, AU: 0.56–0.66, AG: 0.46–0.51; Delta-CoV, AU: 0.53–0.66, AG: 0.46–0.51) after about 620 steps of mutation, and for Beta-CoV (AU: 0.54–0.69, AG: 0.46–0.51) is after around 1100 steps of mutation.

#### 3.2.3. Variation of the Distribution of Stop Codons

To investigate the specificity of mutations on the aspect of sequence permutation, we analyzed the distribution of stop codons which is an important sequence permutation [42,43]. We investigate the permutation patterns associated with four SARS-CoV-2 lineages (Gamma-CoV, Alpha-CoV, Delta-CoV, and Beta-CoV) by interrogating the distribution specificity of new-generated stop codons on reference genome during simulation.

**Figure 7:**
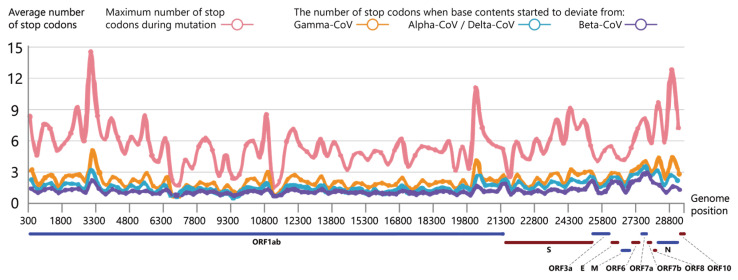
The distribution of new-generated stop codons during mutation simulation. The horizontal axis represents the locations of stop codons on SARS-CoV-2 gene segments and the vertical axis represents the average number of these stop codons within the window length (300 bases). Pink, orange, blue and purple lines represent the maximum number of stop codons during mutation (Method S3), the number of stop codons when base content percentage start to deviate from Gamma-CoV, Alpha-CoV /Delta-CoV and Beta-CoV, respectively.

Figure 7 not only shows the distribution of maximum number of stop codons, which indicates that a new stop codon will generate after about 1 to 15 steps of mutation (about 7 steps on average), but also it demonstrates that each distribution of stop codons we interested have a strong periodic pattern in terms of the overall distribution of the stop codons.

#### 3.2.4. Periodicity of the Distribution of Stop Codons

Since the distribution of stop codons in nucleic acid sequence can be considered as the discrete random signals [44], we investigate the periodic pattern of the distribution of stop codons on the reference genome by power spectrum analysis [44].

**Figure 8:**
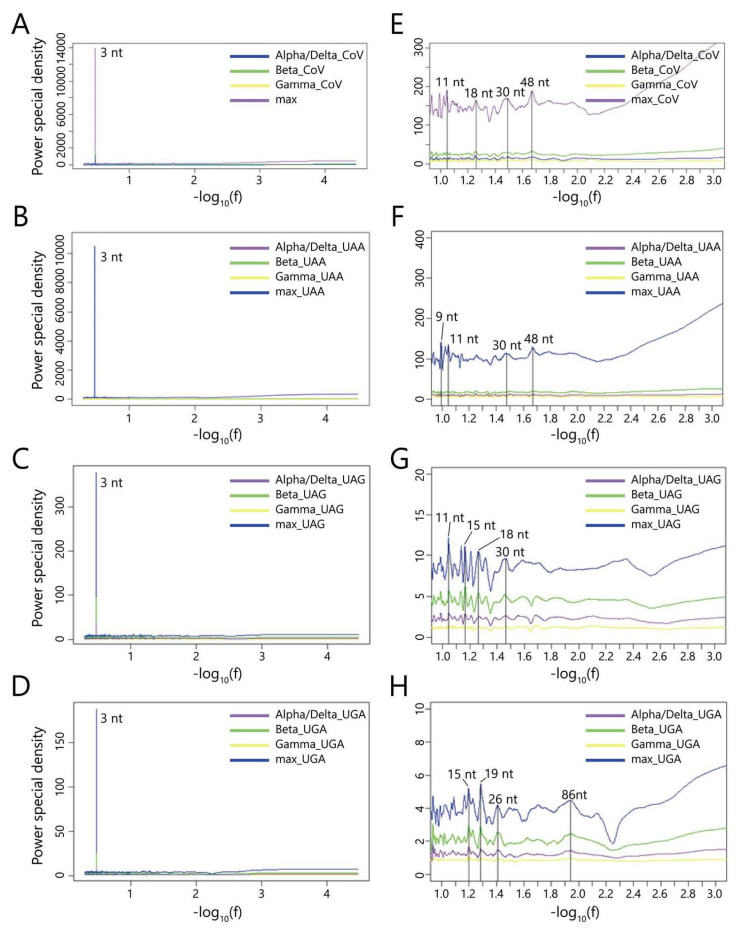
Implementation of sequence periodicity analysis using power spectrum. (**A**) Overall power spectrum of all kinds of stop codons (UAA, UAG and UGA); (**B**) Overall power spectrum of the stop codon UAA; (**C**) Overall power spectrum of the stop codon UAG; (**D**) Overall power spectrum of the stop codon UGA. Since 3 nt frequency peaks in the overall power spectrum (represent stop codons, (**A**–**D**) are obvious, we zoom in each figure to investigate other periodic patterns except for 3 nt by (**E**–**H**). The horizontal axis ($-\log_{10}f$)) represents the length of periodic sequences (f) and the vertical axis represents the power density of corresponding spectrum. Purple, green, and yellow lines of each stop codon represent the power spectrum when the maximum number of stop codons during mutation (Method S3) starts to deviate from the base content percentage of Alpha-CoV/Delta-CoV, Beta-CoV and Gamma-CoV, respectively.

Despite the most obvious 3 nt peak in Figure 8A–E shows that the power spectrum of all kinds of stop codons have peaks at 11 nt, 18 nt, 30 nt and 48 nt. Table 1 demonstrates that periodicity of UAA (Figure 8F) is closer to all kinds of stop codons that illustrated in Figure 8E, which indicates that the periodicity of UAA is much greater than that of UAG and UGA (Figure 8G,H).

### 3.3. Web Service Construction

To address our third scientific question, we establish the SARS-CoV-2 mutation simulation analysis online service platform (http://www.combio-lezhang.online/2019NCSS/home.html, accessed on 22 September 2022, 2019-NCSS), which provides two online services: “mutation spectra analysis” and “mutation simulation”.

2019-NCSS uses Tomcat [26] as the back-end service architecture to enable the surveillance and response to user access. The platform also utilizes Java, C++, and R to implement different back-end computing functions respectively. The front-end uses HTML and JavaScript to implement the web interface, and uses Echarts for data visualization.

**Figure 9:**
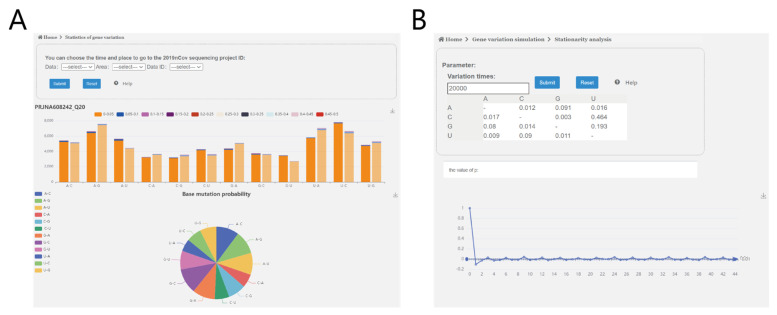
The webpage of 2019-NCSS. (**A**) The “mutation spectra analysis” module. (**B**) The “mutation simulation” module.

Figure 9A shows the “mutation spectra analysis” module with two functions. One is “SARS-CoV-2 strand-specific mutations probability histogram”, which displays the corresponding number of positive- and negative-sense strand base mutants in different base mutation types (Figure S2); And the other is “SARS-CoV-2 mutation spectra”, which can compute and visualize the mutation spectra of positive- and negative-sense strands of a specific project for sequencing data (Figure 5).

Figure 9B shows the “mutation simulation” module with two functions, one is the “dynamics analysis of sequence mutation “, which can analyze the variation of base content percentage during simulation (Figure 6); And the other is the “power spectrum density analysis”, which can analyze the variation of power spectrum density during the simulation (Figure 8).

## 4. Discussion

This study proposes a novel computational analysis algorithm for SARS-CoV-2 genomic mutation spectra based on nanopore sequencing and nucleobase filtering as well as accurately computes the intra-host mutation spectra of positive- and negative-sense strands within different lineages of SARS-CoV-2. Then, we not only build up a novel Markov chain-based intra-host mutation simulation model, but also analyze different sequence properties such as base content percentage, the distribution of stop codons and sequence periodicity of the SARS-CoV-2 mutation spectra during mutation. Finally, we establish an online service platform for SARS-CoV-2 mutation spectra analysis and mutation simulation, which provide researchers with data downloading, online computational analysis and visualization services.

For our first scientific question: how to develop such a data specificity-based base filtering algorithm for SARS-CoV-2 sequencing data that can significantly improve the accuracy of strand-specific mutation spectra computation for SARS-CoV-2, we found that the average base quality processed by the nucleobase filtering algorithm with dynamic threshold is statistically better than the other four classical methods, indicating the effectiveness of our base filtering algorithm. Meanwhile, the major mutations on the positive-sense strand of our mutation spectra are consistent with that discovered by aligned genomes before (much higher mutation rate of C->U than U->C and G->U than U->G [45]). Furthermore, the intra-host mutation spectra shows that there is a statistically significant difference between each probability of base mutation types in positive- and negative-sense strand, indicating that the computational method we developed can effectively split strand-specific SARS-CoV-2 sequencing reads, and analyze the intra-host mutations which may be covered by aligned genomes [46] or not accumulated into the viral population [47].

For our second scientific question: how to develop an efficient SARS-CoV-2 intra-host mutation simulation and data analysis algorithm with respect to the intra-host mutation spectra and coronavirus mutation characteristics, Figure S3 indicates that the simulation efficiency can be significantly increased by determining the upper limit of the simulation steps. Next, the variation of base content during simulation reveals that the simulation sequence starts to deviate from the base content percentage of Alpha-CoV/Delta-CoV after approximately 620 steps of mutation. This finding is consistent with previous studies, in which a cell generates approximately 600 to 700 infectious units on average [16], indicating that viral particles are more likely to be released continuously rather than produced by a one-off cell lysis [48]. Moreover, we demonstrate that a new stop codon will be generated after seven steps of mutation on average, which is very close to the average mutation accumulation rate per patient (half a dozen mutations on average [27]). The two above results, which are correspond with previous studies, demonstrate the validity of our mutation simulation method.

Besides, we found that the periodicity of UAA is much greater than that of UAG and UGA. On the one hand, this agrees with our results in the histogram Figure S2, in which the base mutation types A->U and U->A have the highest number of mutants, therefore the mutated genome is more likely to generate new stop codons of UAA; on the other hand, this suggests the presence of the sequence alignment preference in intra-host mutations generated during genome synthesis [47]. These results may provide new evidences for the further investigation of the complex process that how a single viral mutation accumulates and is inherited by all offspring to generate viral lineages.

For our third scientific question: how to build up a visualized web service platform for online mutation spectra analysis and simulation, our web service 2019-NCSS can not only provide researchers with data downloading, online computational analysis and visualization services, but also allow validation and feedback optimization of the mutation simulation process using continuously accumulated data.

In summary, this study investigates the intra-host mutation of SARS-CoV-2 in terms of strand-specific mutation spectra computation, mutation simulation analysis and online service development. However, since SARS-CoV-2 is still mutating and threatening human health as well as 2019-NCSS do not support online real-time computing for each function due to the limited computing power, it is still urgent for us to develop the new prediction method for the future mutation risk of SARS-CoV-2 by combining our current study with the transmissibility and pathogenicity of mutated virus with high-performance computing methods [4,31,34,38].

## Figures and Tables

**Table 1:** Periodicity of the distribution of three stop codons.

Stop Codons	The Length of Segment Corresponding to the Most Significant Peak Other Than 3 nt
UAA	9/11/30/48 nt
UAG	11/15/18/30 nt
UGA	15/19/26/86 nt

## Data Availability

Data presented in this study are available in the Materials and Methods section and Supplementary Materials.

## Supplementary Materials

The following supporting information can be downloaded at: https://www.mdpi.com/article/10.3390/biom13010063/s1, Method S1: The validation of nucleobase filtering algorithm with dynamic threshold [49,50,51]; Method S2: The computation of mutation probability matrix by nucleobase mutation probabilities; Method S3: The computation of maximum number of stop codons during mutation; Figure S1: The pseudo code of intra-host mutation spectra computation; Figure S2: The histogram of SARS-CoV-2 strand-specific mutations probability; Figure S3: Verifying the upper limit of the mutation using information entropy; Table S1: Data sources that used in this paper; Table S2: The group settings for the test of significance; Table S3: Results of the normality test and the test of significance for four groups of SARS-CoV-2 positive-sense strand; Table S4: Results of the normality test and the test of significance for four groups of SARS-CoV-2 negative-sense strand; Table S5: Computed mutation probability of nucleobases in our previous research.
