# Supplementary material for: A Computer Simulation of SARS-CoV-2 Mutation Spectra for Empirical Data Characterization and Analysis
Source: Biomolecules. 2022 Dec 28;13(1):63. doi: 10.3390/biom13010063 (PMC9855923; doi:10.3390/biom13010063)
Supplement: Supplementary file 1 [file biomolecules-13-00063-s001.zip › biomolecules-2100334-supplementary.pdf]

SUPPLEMENTAL INFORMATION

Supplementary Methods

Method S1. The validation of nucleobase filtering algorithm with dynamic threshold.

Method S2. The computation of mutation probability matrix by nucleobase mutation probabilities.

Method S3. The computation of maximum number of stop codons during mutation.

Supplementary Figures

Figure S1. The pseudo code of intra-host mutation spectra computation.

Figure S2. The histogram of SARS-CoV-2 strand-specific mutations probability.

Figure S3. Verifying the upper limit of the mutation using information entropy.

Supplementary Tables

Table S1. Data sources that used in this paper.

Table S2. The group settings for the test of significance.

Table S3. Results of the normality test and the test of significance for four groups of SARS-CoV-2 positive-sense strand.

Table S4. Results of the normality test and the test of significance for four groups of SARS-CoV-2 negative-sense strand.

Table S5. Computed mutation probability of nucleobases in our previous research.

Supplementary Methods

Method S1. The validation of nucleobase filtering algorithm with dynamic threshold

The base quality score (Phred quality score) is widely used to measure the effectiveness of filtering algorithms. This study uses an approach that based on comparing the average base quality score [1] to verify the effectiveness of dynamic threshold base filtering algorithms. We use **Eq. S1** to compare the average base quality score at each site on the positive- and negative-sense reference genome of our dynamic filtering algorithm with several commonly used algorithms (unfiltered, seqtk, fastp, sickle).

$$Q_{average} = \frac{\sum_{i=1}^{n} Q_i}{n} \tag{S1}$$

Here, n represents the total number of bases at each site, Q_i represents the base quality of base i, $i = 1,2,\ldots,n$.

Next, we use Shapiro-Wilk test [2] **(Eq. S2)** to test the normality of the computed results.

$$W = \frac{\left(\sum_{i=1}^{n} a_i x_i\right)^2}{\sum_{i=1}^{n}(x_i - \overline{x})^2} \tag{S2}$$

Here, x_i represents the i th order statistic; $\overline{x}$ represents the sample mean; the coefficients a_i are given by vector $(a_1,\ldots,a_n) = m^T V^{-1}/(m^T V^{-1} V^{-1} m)^{1/2}$, where vector $m = (m_1,\ldots,m_n)^T$ is the expected vector of the order statistics of independent and identically distributed random variables sampled from the standard normal distribution, and V is the covariance matrix of these normal order statistics. If the test result of the sample is less than the chosen alpha level, then there is evidence that the sample are not normally distributed [3].

Subsequently, we use the Mann-Whitney U test [4] to test the significance of difference between the base quality score on reference genome that processed by the nucleobase filtering algorithm with dynamic threshold and the other four filtering methods (group settings can be referred in **Table S2**). Accordingly, we mix two sets of sample data and assign numeric ranks in ascending order to the mixed data, where the smallest data is assigned by 1 and the second smallest data by 2, etc. Where there are groups of tied data, we assign ranks that equal to the mean value of their unadjusted ranks. Then, we compute the sum of ranks in two sets T_1 and T_2, respectively. Finally, the Mann-Whitney U test is performed on the two sets of data by **Eq. S3**.

$$Z = \frac{U - \frac{n_1 n_2}{2}}{\sqrt{\frac{n_1 n_2 (n_1 + n_2 + 1)}{12}}} \quad \text{(S3)}$$

Here, $U = \min\{\frac{n_1 n_2 + n_1(n_1+1)}{2} - T_1, \frac{n_1 n_2 + n_2(n_2+1)}{2} - T_2\}$, where n_1 and n_2 is the size for sample 1 and 2, respectively, T_1 and T_2 is the sum of the ranks in sample 1 and 2, respectively. If the test result of sample 1 and 2 is less than the chosen alpha level, then there is evidence that the sample 1 and 2 is statistically different.

The results of the test of significance are listed in **Table S3** and **S4**. It is clearly that that the nucleobase filtering algorithm with dynamic threshold is statistically effective to filter low-quality sequencing data than other four algorithms.

Method S2. The computation of mutation probability matrix by nucleobase mutation probabilities

The computation of the mutation probability matrix $P_{i,j} = \{p_{i,j}\}$ （**Eq. 6**） is comprised of two scenarios: locating the mutation probability term r_i for each base i **(Eq. 4)** and the probability term $m_{i,j}$ that base i mutates to base j in the mutation spectra.

The n_i term in r_i can be computed from **Eq. 5**, where $mutation\_rate = 10^{-4}$ [5] and $geo\_len \approx 3 \times 10^{-4}$ (length of the SARS-CoV-2 reference genome) for base i; the n_{all} term is the length of the coding region on SARS-CoV-2 reference genome, which has 29260 bases in total [6]. Therefore, $r_i = r_A = r_U = r_C = r_G$ =3/29260. In addition, the $m_{i,j}$ is from our previous research **(Table S5)** [7]. The above r_i and $m_{i,j}$ values are inputted into the mutation probability matrix **(Eq. 6)** to compute its value (**Eq. 10**).

Method S3. The computation of maximum number of stop codons during mutation

In order to investigate whether viral genomic sequence have a long-term accumulation of the distribution of stop codons, we carry out mutation simulation according to **Methods 2.2.1** and computed the number of stop codons within each window length (300 bases) after each step of simulation $Num\_SC(step, win\_len)$. We then calculated the number of stop codons within each window length in that simulation where the maximum number of stop codons occurred during the mutation by **Eq. S4.**

$$MaxNum\_SC(win\_len) = \max\left[Num\_SC(step, win\_len)\right] \tag{S4}$$

Next, to reduce the randomness caused in one time of simulation, we repeat the simulation $run\_times = 100$ times, and then we compute average $MaxNum\_SC(win\_len)$ by **Eq. S5**

$$Aver\_MaxNum\_SC(win\_len) = \frac{\sum_{run\_times} MaxNum\_SC(win\_len)}{run\_times} \tag{S5}$$

Finally, we investigate the distribution of stop codons at each site on the reference genome during mutation with $Aver\_MaxNum\_SC(win\_len)$ as the maximum number of stop codons during mutation **(Figure 6)**.

Supplementary Figures

Input: The reference sequence, Dictionary $D = \{'quality\_scores': base\_numbers\}$ at each site, Nanopore sequencing reads data

Output: Mutation spectrum of 12 mutation directions

Mapping each sequencing data reads to the reference genome
for each reference genome site
    D is sorted by $'quality\_score'$ in ascending order
    Let $left = 1$, $right = size(D)$; $S_{left} = Q_{left} * N_{left}$ and $S_{left} = Q_{left} * N_{left}$
    while $left \leq right$ **do**
        if $S_{left} < S_{right}$ **then**
            $left++$, $S_{left} = S_{left} + Q_{left} * N_{left}$
        else if $S_{left} > S_{right}$ **then**
            $right--$, $S_{right} = S_{right} + Q_{right} * N_{right}$
        else
            $left++, right--, S_{left} = S_{left} + Q_{left} * N_{left}, S_{right} = S_{right} + Q_{right} * N_{right}$
        end if
    end while
    all bases with quality scores less than threshold Q_{left} are filtered out
end for
for each reference genome site
    for each sequencing data reads after filter
        if (genome site base==r and base of reads==m)
            $p_{site}(r \to m)$++
        end if
    end for
end for
for each variant direction (12 in total)
    return $p_{genome}(r \to m) = total\ number\ of\ p_{site}(r \to m) / n(r \to m)$
end for

Figure S1 The pseudo code of intra-host mutation spectra computation. Here, D represents the dictionary which stores $'quality\_scores'$: $base\_numbers$ pairs at each site; V represents the base dataset after filtering by the algorithm at each site; Q represents the base quality score corresponding to $left$ or $right$; N represents the base number corresponding to Q_{left} or Q_{righ}. and $p_{site}(r \to m)$ represents the probability of base mutation type $r \to$

m at each site, $n(r \rightarrow m)$ represents the number of sites where base mutation type $r \rightarrow m$ occurs. We implemented the algorithm separately for the positive and negative strand sequencing datasets to obtain the intra-host mutation spectra results for the positive- and negative-sense sub-genomes, respectively.

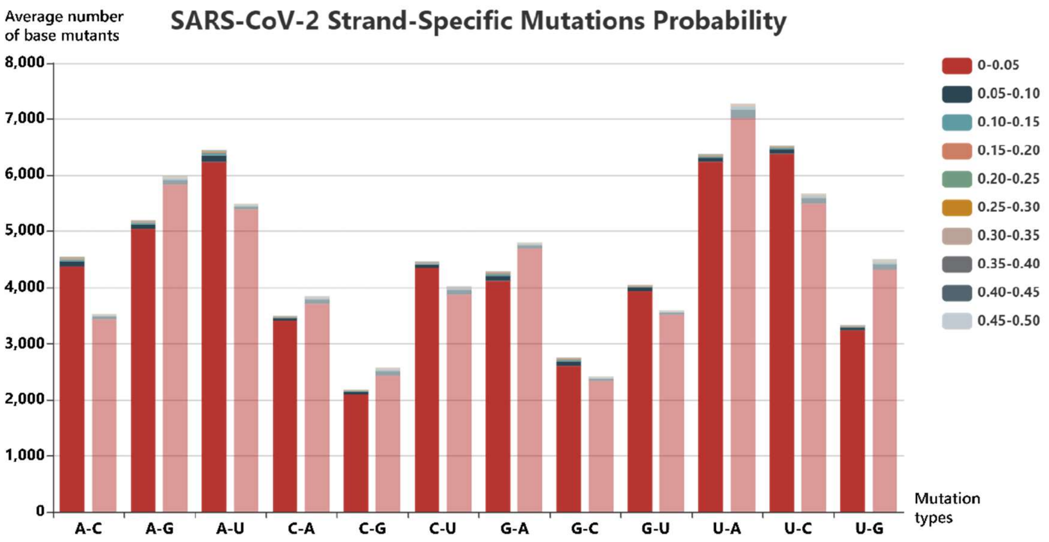

Figure S2. The histogram of SARS-CoV-2 strand-specific mutations probability. Here, the height of each column represents the sum of corresponding number of positive- and negative-sense strand base mutants in different base mutation types. The left column of the mutation types (the deep red colored) represents the positive-sense strand while the right (light red colored) represents the negative-sense strand, and the color on the top of each column represents the probability of each base mutation type occurs.

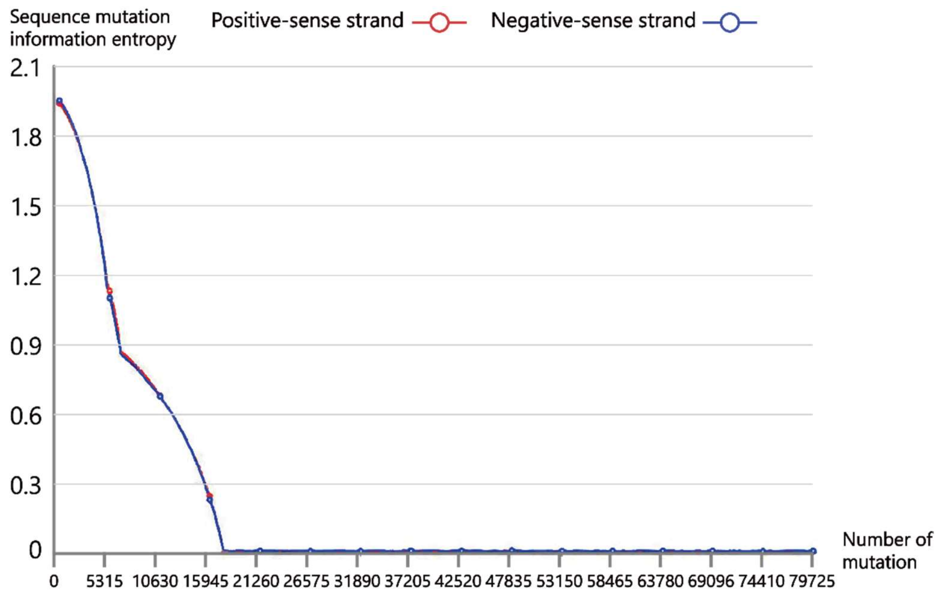

Figure S3. Verifying the upper limit of the mutation using information entropy. As the steps of mutation simulation increase, the content of each base varies and eventually tends to be constant **(Figure 5)**. The result of this process is the decrease of uncertainty on the whole sequence; thus, information entropy can be used to measure the uncertainty and determine the upper limit of the mutation **(Section 3.2.2)**. Here, we find that the information entropy of the positive- and negative-sense strand converges to stationary distribution after about 80,000 steps. Therefore, we can greatly increase the efficiency of the SARS-CoV-2 intra-host mutation simulation by determining the upper limit of the mutation.

Supplementary Tables

Table S1. Data sources that used in this paper

Data Name	Original Source	Download Link
SARS-CoV-2 nanopore sequencing data	NCBI SRA	https://github.com/FuboMa/DoctorProjects/tree/master/Project%20SARS-CoV-2/Data%20directory

Table S2. The group settings for the test of significance

Group Number	Setting
1	$Q_{average}$ at each site under **unfiltered** or **nucleobase filtering algorithm with dynamic threshold**
2	$Q_{average}$ at each site under **fastp** or **nucleobase filtering algorithm with dynamic threshold**
3	$Q_{average}$ at each site under **seqtk** or **nucleobase filtering algorithm with dynamic threshold**
4	$Q_{average}$ at each site under **sickle** or **nucleobase filtering algorithm with dynamic threshold**

Table S3. Results of the normality test and the test of significance for four groups of SARS-CoV-2 positive-sense strand.

Positive-sense Strand	Group 1		Group 2		Group 3		Group 4	
	unfil.	d. t.	fastp	d. t.	seqtk	d. t.	sickle	d. t.
**p-value of S. W. test**	0	0	0	0	0	0	0	0
**normalized?**	*N*	*N*	*N*	*N*	*N*	*N*	*N*	*N*
**p-value of M. W. U test**	0		0		0		0	
**difference significant?**	*Y*		*Y*		*Y*		*Y*	

Table S4. Results of the normality test and the test of significance for four groups of SARS-CoV-2 negative-sense strand.

**Negative-sense Strand**	**Group 1**		**Group 2**		**Group 3**		**Group 4**	
	unfil.	d. t.	fastp	d. t.	seqtk	d. t.	sickle	d. t.
**p-value of S. W. test**	0	0	0	0	0	0	0	0
**normalized?**	*N*	*N*	*N*	*N*	*N*	*N*	*N*	*N*
**p-value of M. W. U test**	0		0		0		0	
**difference significant?**	*Y*		*Y*		*Y*		*Y*	

Table S5. Computed mutation probability of nucleobases in our previous research [7].

**To** / **Mutate from**	**A**	**U**	**C**	**G**
**A**	-	0.137	0.102	0.761
**U**	0.084	-	0.817	0.099
**C**	0.034	0.959	-	0.007
**G**	0.278	0.673	0.049	-

Reference

1. Andrews, S. FastQC A Quality Control tool for High Throughput Sequence Data. Available online: http://www.bioinformatics.babraham.ac.uk/projects/fastqc/ (accessed on 1 August 2018).
2. Hanusz, Z.; Tarasinska, J.; Zielinski, W. Shapiro-Wilk te st with known mean. *REVSTAT-Stat. J.* **2016**, *14*, 89–100.
3. Schneider, J.W. Null hypothesis significance tests. A mix-up of two different theories: The basis for widespread confusion and numerous misinterpretations. *Scientometrics* **2015**, *102*, 411–432.
4. Boyce, E.G.; Nappi, J.M. Is there significance beyond the t-test. *Drug Intell Clin Pharm* **1988**, 22, 334-335
5. Teng, X.; Li, Q.; Li, Z.; Zhang, Y.; Niu, G.; Xiao, J.; Yu, J.; Zhang, Z.; Song, S. Compositional variability and mutation spectra of monophyletic SARS-CoV-2 clades. *Genom. Proteom. Bioinform.* **2020**, *18*, 648–663.
6. Park, W.B.; Kwon, N.-J.; Choi, S.-J.; Kang, C.K.; Choe, P.G.; Kim, J.Y.; Yun, J.; Lee, G.-W.; Seong, M.-W.; Kim, N.J. Virus isolation from the first patient with SARS-CoV-2 in Korea. *J. Korean Med. Sci.* **2020**, *35*, e84. https://doi.org/10.3346/jkms.2020.35.e84.
7. Yu, J. From Mutation Signature to Molecular Mechanism in the RNA World: A Case of SARS-CoV-2. *Genom. Proteom. Bioinform.* **2020**, *18*, 627–639. https://doi.org/10.1016/j.gpb.2020.07.003.
